# Transposon mutagenesis and identification of mutated genes in growth-delayed *Edwardsiella ictaluri*

**DOI:** 10.1186/s12866-019-1429-3

**Published:** 2019-03-08

**Authors:** Safak Kalindamar, Jingjun Lu, Hossam Abdelhamed, Hasan C. Tekedar, Mark L. Lawrence, Attila Karsi

**Affiliations:** Department of Basic Sciences, College of Veterinary Medicine, Mississippi State University, Mississippi State, MS USA

**Keywords:** p*MAR2xT7*, Enteric septicemia, Catfish, Virulence, Type III secretion system

## Abstract

**Background:**

*Edwardsiella ictaluri* is a Gram-negative facultative intracellular anaerobe and the etiologic agent of enteric septicemia of channel catfish (ESC). To the catfish industry, ESC is a devastating disease due to production losses and treatment costs. Identification of virulence mechanisms of *E. ictaluri* is critical to developing novel therapeutic approaches for the disease. Here, we report construction of a transposon insertion library and identification of mutated genes in growth-delayed *E. ictaluri* colonies. We also provide safety and efficacy of transposon insertion mutants in catfish.

**Results:**

An *E. ictaluri* transposon insertion library with 45,000 transposants and saturating 30.92% of the TA locations present in the *E. ictaluri* genome was constructed. Transposon end mapping of 250 growth-delayed *E. ictaluri* colonies and bioinformatic analysis of sequences revealed 56 unique *E. ictaluri* genes interrupted by the *MAR2xT7* transposon, which are involved in metabolic and cellular processes and mostly localized in the cytoplasm or cytoplasmic membrane. Of the 56 genes, 30 were associated with bacterial virulence. Safety and vaccine efficacy testing of 19 mutants showed that mutants containing transposon insertions in hypothetical protein (*Ei*s::*004*), and Fe-S cluster assembly protein (IscX, *Ei*s::*039*), sulfurtransferase (TusA, *Ei*s::*158*), and universal stress protein A (UspA, *Ei*s::*194*) were safe and provided significant protection (*p* < 0.05) against wild-type *E. ictaluri*.

**Conclusions:**

The results indicate that random transposon mutagenesis causing growth-delayed phenotype results in identification bacterial virulence genes, and attenuated strains with transposon interrupted virulence genes could be used as vaccine to activate fish immune system.

## Background

Enteric septicemia of catfish (ESC) is a devastating disease that causes significant production loss and treatment cost for the catfish aquaculture industry [1]. A few antimicrobials and a commercial live attenuated vaccine are available for treatment of ESC. However, treatment of sick catfish by medicated feed is not effective due to early onset of anorexia. The extensive use of antimicrobials can induce the appearance of resistant strains [2, 3]. The commercial ESC vaccine Aquavac-ESC has been available for the catfish industry for more than 15 years [4], but ESC is still one of the major diseases in the US catfish industry.

*Edwardsiella ictaluri* is well-adapted to channel catfish [5, 6] and some of the *E. ictaluri* virulence factors include lipopolysaccharide (LPS), flagella, outer membrane proteins (OMPs), and extracellular proteins [7–10]. There have been several reports on development of attenuated *E. ictaluri* strains by deleting genes involved in iron acquisition, tricarboxylic acid cycle, one-carbon metabolism, and amino acid biosynthesis [11–17]. However, virulence mechanisms of *E. ictaluri* are not understood well, and there is a need for identification of novel virulence-related genes to develop effective live attenuated vaccines.

Random transposon insertion is a high-throughput genetic manipulation tool that allows random mutation of genes at the genome level. Mariner family transposon *Himar1* inserts itself randomly into “TA” nucleotide sequences [18, 19]. Mariner family transposons have been widely used to generate random mutagenesis in fish pathogen *Mycobacterium marinum,* and also human pathogens such as *Pseudomonas aeruginosa*, *Campylobacter jejuni*, *Leptospira interrogans*, and *Rickettsia prowazekii* [20–24].

In this research, *MAR2xT7* transposon, *a Himar1* derivative [20], was used to identify genes required for *E. ictaluri* growth on a solid complex medium. We expect that colonies exhibiting attenuated growth on solid media will have transposon insertions in important bacterial genes, and these mutants may also show attenuated virulence in the catfish host and potentiate catfish immune responses [14]. Therefore, attenuation and vaccine efficacy of 19 transposon mutants were evaluated in channel catfish.

## Results

### Transposon insertion library

**Fig. 1:**
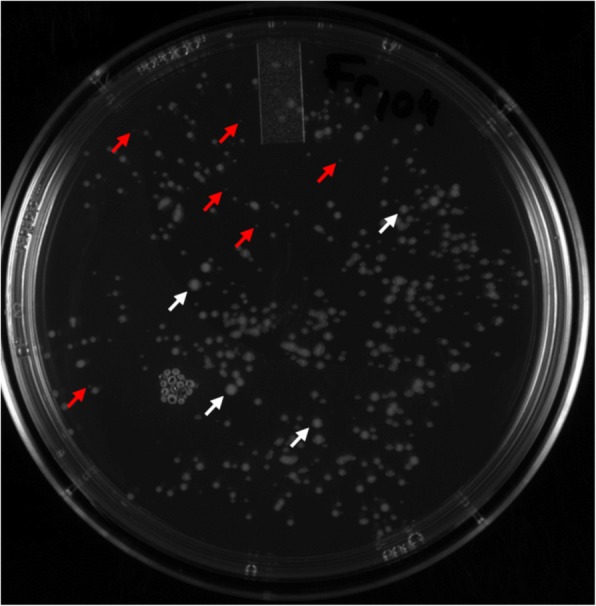
BHI agar plate showing transposon insertion colonies with delayed growth (red arrows) and normal size (white arrows) colonies after 48 h of incubation at 30 °C

By using *MAR2xT7* transposon, an *E. ictaluri* transposon insertion library containing 45,000 transposants was constructed. Colonies with transposon insertion and delayed growth were observed on the BHI agar media after 48 h (Fig. 1). The initial overnight growth of these small colonies in BHI broth was also very slow compared to wild type, but this difference disappeared in later broth cultures (data not shown).

The complete genome size of *E. ictaluri* strain 93–146 is 3,812,301 bp, which contains 3597 total genes. The number of TA locations in the entire *E. ictaluri* genome is 145,515. Thus, 45,000 transposants would saturate 30.92% of the potential *MAR2xT7* transposon insertion sites available. The number of TA locations in the *E. ictaluri* open reading frames is 110,373, which represent 75.85% of all available *MAR2xT7* transposon insertion sites. Thus, random insertion events would saturate 23,45% of the potential *MAR2xT7* transposon insertion sites in the *E. ictaluri* open reading frames.

### Gene identification

Transposon end amplification by single primer PCR yielded 151 samples with PCR products, of which 94 were sequenced successfully. After analysis, 56 unique genes containing transposon insertions were identified (Table 1). These unique genes contained a total number of 2235 *MAR2xT7* transposon insertion sites, and the exact number of *MAR2xT7* transposon insertion site in each gene was indicated in Table 1.

**Table 1:** Unique *E. ictaluri* genes with transposon insertion

Mutant	Locus	Product	E-value^a^	Frequency^b^	TA Frequency
Eis001	NT01EI_1281	NAD-dependent malic enzyme (NAD-ME)	2.00E-72		64
Eis002	NT01EI_1721	PTS system, mannose/fructose/sorbose family, IIB component	2.00E-106		32
Eis004	NT01EI_0182	Hypothetical protein	7.00E-57		26
Eis006	NT01EI_0085	ATP-dependent DNA helicase Rep	2.00E-114		46
Eis009	NT01EI_1236	Coproporphyrinogen III oxidase, aerobic	6.00E-32		24
Eis011	NT01EI_3690	ABC transporter, periplasmic amino acid binding protein	1.00E-74		50
Eis013	NT01EI_2795	Translocator protein, LysE family	2.00E-115		36
Eis018	NT01EI_0377	Aspartate ammonia-lyase	7.00E-145		59
Eis024	NT01EI_3505	Dihydrouridine synthase Dus	1.00E-129		59
Eis027	NT01EI_0408	tRNA delta(2)-isopentenylpyrophosphate transferase	5.00E-94		22
Eis028	NT01EI_0277	Transposase, IS4 family protein	1.00E-73	4	17
Eis029	NT01EI_2683	Membrane protein	2.00E-88		27
Eis033	pEI2_p2	Putative Rep protein	2.00E-123	5	31
Eis035	pEI1_p4	Putative RNA one modulator protein	9.00E-19		12
Eis037	NT01EI_2355	eseJ	3.00E-129	2	88
Eis038	NT01EI_1334	eseM	5.00E-67	2	91
Eis039	NT01EI_3177	FeS assembly protein IscX	3.00E-38		7
Eis041	NT01EI_0943	eseC	8.00E-116		53
Eis048	NT01EI_3148	Hypothetical protein	4.00E-80		20
Eis055	NT01EI_2314	Prophage lambda integrase	8.00E-128		56
Eis059	NT01EI_1941	Hypothetical protein	3.00E-70		8
Eis065	NT01EI_2281	Excinuclease ABC subunit C	3.00E-48		64
Eis068	NT01EI_0448	Polyprenyl synthetase	1.00E-115		37
Eis080	NT01EI_0981	Hypothetical protein	8.00E-129	9	58
Eis086	NT01EI_1237	N-acetylmuramoyl-L-alanine amidase AmiA	3.00E-67		25
Eis107	NT01EI_0475	DEAD box containing helicase	1.00E-112		51
Eis110	NT01EI_1332	eseL	4.00E-26	2	73
Eis131	NT01EI_2157	Hypothetical protein	4.00E-66		37
Eis152	NT01EI_0224	Transporter, major facilitator family	1.00E-16	4	47
Eis154	NT01EI_3522	Selenate reductase, FAD-binding subunit	2.00E-73		23
Eis155	NT01EI_0725	Transcriptional regulator FruR	3.00E-134		43
Eis156	NT01EI_2381	Ribonuclease, RNaseE/RNaseG family	2.00E-04		72
Eis157	NT01EI_0144	Twin-arginine translocation protein subunit TatB	8.00E-07		13
Eis158	NT01EI_0022	Sulfurtransferase, TusA	8.00E-46		10
Eis171	NT01EI_3723	Magnesium-translocating P-type ATPase	0		71
Eis172	NT01EI_3786	Hypothetical protein	1.00E-26		24
Eis173	NT01EI_3265	Acyltransferase/AMP-dependent synthetase and ligase family	0	2	54
Eis174	NT01EI_3721	Hypothetical protein	3.00E-25		7
Eis175	NT01EI_3774	IS1 transposase	7.00E-93		19
Eis176	NT01EI_0962	esaT	5.00E-129		27
Eis180	NT01EI_3103	UPF0126 domain protein	7.00E-12		16
Eis183	NT01EI_3105	Chloride transporter, chloride channel (ClC) family	6.00E-166		53
Eis184	NT01EI_0419	RNA methyltransferase, TrmH family, group 3	9.00E-27		21
Eis185	NT01EI_3386	TRAP transporter, DctM subunit	7.00E-85		47
Eis192	NT01EI_3147	Hypothetical protein	0	2	103
Eis194	NT01EI_1981	Universal stress protein A uspA	9.00E-86	3	20
Eis195	NT01EI_0376	Anaerobic C4-dicarboxylate transporter DcuA	2.00E-159	11	47
Eis207	NT01EI_1817	Spermidine/putrescine transport system permease protein PotB	4.00E-132		22
Eis210	NT01EI_0800	Prolipoprotein diacylglyceryl transferase	5.00E-79		36
Eis220	NT01EI_2076	Hypothetical protein	6.00E-20		5
Eis222	NT01EI_0768	Hypoxanthine phosphoribosyltransferase	5.00E-127		24
Eis223	NT01EI_1086	Extracellular solute-binding protein, family 5	2.00E-142		48
Eis230	NT01EI_3769	Phosphoglycerate transporter family protein	0		49
Eis232	NT01EI_2010	Hypothetical protein	2.00E-136	3	58
Eis233	NT01EI_2530	Putative permease, membrane region	4.00E-81		65
Eis235	NT01EI_3289	Diaminopimelate decarboxylase	9.00E-36		38

^a^Blastx E-value

^b^p*MAR2xT7* insertion frequency

### Functional annotation

**Fig. 2:**
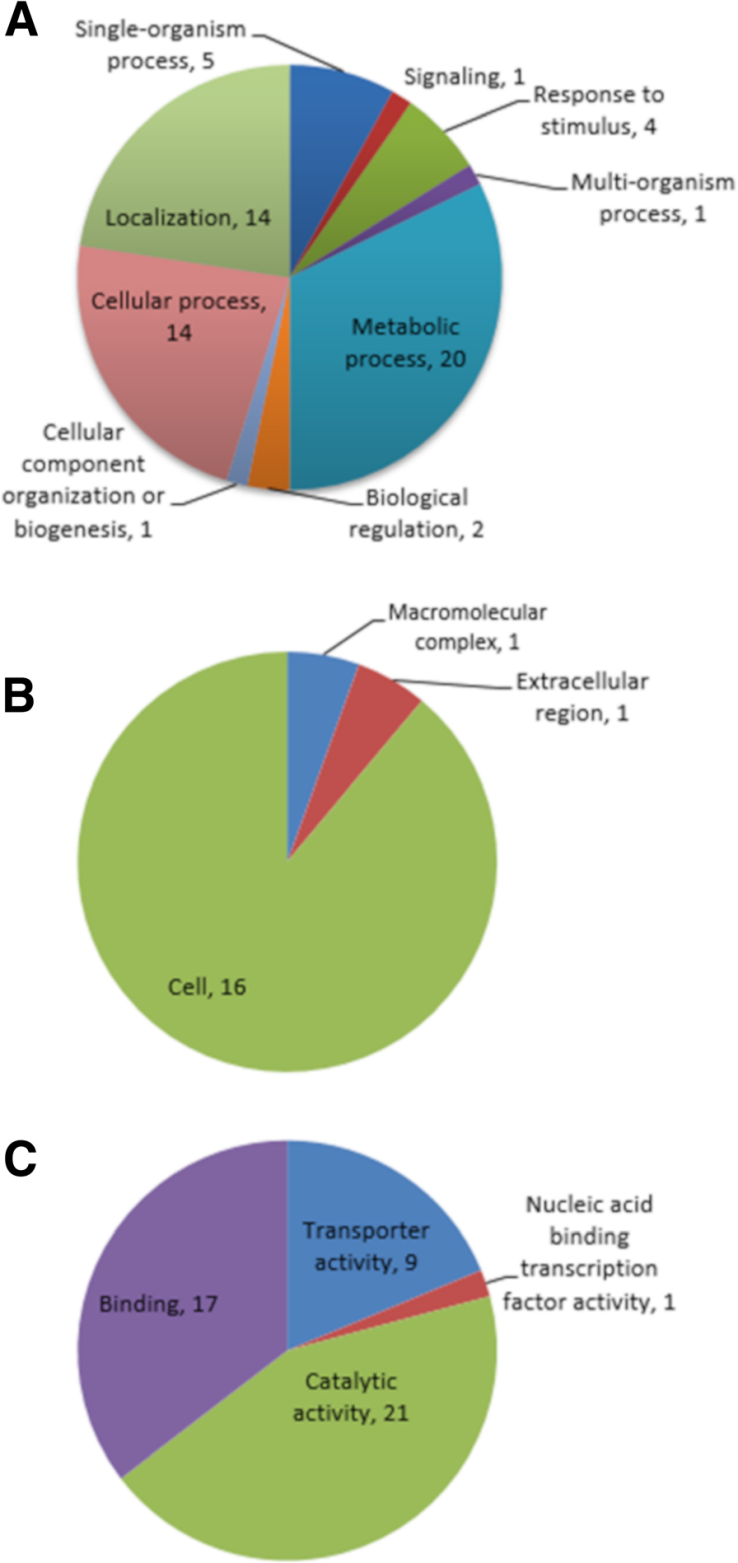
Gene ontology (GO) analysis of *E. ictaluri* transposon inserted genes. GO terms at level 2 according to biological process (**a**), GO terms at level 2 according to cellular component (**b**), GO terms at level 2 according to molecular function (**c**)

Protein sequences of all 56 genes were annotated functionally and assigned to biological process (localization, cellular process, metabolic process, response to stimulus, biological regulation, signaling, multi-organism process, single-organism process, and biogenesis), cellular component (cell, macromolecular complex, and extracellular region), and molecular function (binding, transporter activity, catalytic activity, and nucleic acid binding transcription factor) (Fig. 2).

### Subcellular localization

The locations of 15 proteins were unknown. Of the 41 proteins with known subcellular location, most were localized to the cytoplasm (20 proteins) and cytoplasmic membrane (16 proteins). Extracellular space, outer membrane, and periplasm contained very few proteins (3, 1, 1 proteins, respectively).

### Proteins involved in host-pathogen interactions

Out of 56 identified unique proteins, 30 proteins had significant homology to Host-Pathogen Interaction Database (HPIDB) (Table 2). The proteins mostly matched to the *Enterobacteriaceae* (*Yersinia pestis*, *Escherichia coli K12, Shigella flexneri*)*, Francisellaceae* (*Francisella tularensis SCHU S4*)*,* and *Bacillaceae* families (*Bacillus anthracis*).

**Table 2:** Genes involved in host-pathogen interactions

Mutant	Accession Nu.	Protein	Organism	E-value
Eis152	YP_019321.1	Oxalate:formate antiporter, putative	*Bacillus anthracis*	2.00E-20
Eis155	YP_017710.1	Sugar-binding transcriptional regulator, LacI family	*Bacillus anthracis*	2.00E-21
Eis207	YP_002347936.1	Inner membrane permease T of sulfate/thiosulfate ABC transporter	*Yersinia pestis*	1.00E-12
Eis011	YP_017492.1	Amino acid ABC transporter, amino acid-binding protein	*Bacillus anthracis*	1.00E-15
Eis013	NP_670988.1	Putative threonine efflux protein	*Yersinia pestis*	5.00E-11
Eis171	YP_002345523.1	Putative cation transport protein	*Yersinia pestis*	4.00E-88
Eis223	YP_002345598.1	HTH-type transcriptional regulator SgrR	*Yersinia pestis*	2.00E-46
Eis176	NP_857736.1	Yop proteins translocation protein T	*Yersinia pestis*	9.00E-31
Eis107	YP_022388.1	ATP-dependent RNA helicase, DEAD/DEAH box family	*Bacillus anthracis*	1.00E-84
Eis024	NP_842644.2	tRNA-dihydrouridine synthase	*Bacillus anthracis*	6.00E-62
Eis110	NP_858359.2	E3 ubiquitin-protein ligase ipaH9.8	*Shigella flexneri*	6.00E-90
Eis086	NP_667964.1	N-acetylmuramoyl-l-alanine amidase II	*Yersinia pestis*	1.00E-39
Eis184	YP_016695.1	RNA methyltransferase, TrmH family, group 3	*Bacillus anthracis*	6.00E-43
Eis180	YP_002345138.1	Putative membrane protein	*Yersinia pestis*	4.00E-20
Eis006	YP_170066.1	ATP-dependent DNA helicase	*Francisella tularensis*	1.00E-171
Eis009	YP_170044.1	Coproporphyrinogen-III oxidase, aerobic	*Francisella tularensis*	9.00E-52
Eis027	YP_169650.1	tRNA dimethylallyltransferase	*Francisella tularensis*	8.00E-76
Eis041	YP_002345337.1	Possible type III secretion protein	*Yersinia pestis*	4.00E-154
Eis173	NP_994169.1	Bifunctional protein aas	*Yersinia pestis*	0
Eis235	YP_002345851.1	Diaminopimelate decarboxylase	*Yersinia pestis*	7.00E-177
Eis156	NP_669066.1	RNase E	*Yersinia pestis*	0
Eis183	NP_668136.1	H(+)/Cl(−) exchange transporter ClcA	*Yersinia pestis*	0
Eis065	NP_669748.1	UvrABC system protein C	*Yersinia pestis*	0
Eis002	YP_002346757.1	PTS enzyme IIAB, mannose-specific	*Yersinia pestis*	3.00E-149
Eis222	YP_646612.1	Hypoxanthine phosphoribosyltransferase	*Yersinia pestis*	6.00E-84
Eis068	YP_491372.1	Octaprenyl-diphosphate synthase	*Escherichia coli*	2.00E-157
Eis001	YP_002346527.1	NAD-dependent malic enzyme	*Yersinia pestis*	0
Eis230	YP_001608410.1	Putative regulatory protein	*Yersinia pestis*	0
Eis018	NP_667943.1	Aspartate ammonia-lyase (Aspartase)	*Yersinia pestis*	0
Eis233	YP_002346351.1	Putative transport protein YPO1326/y2857/YP_1266	*Yersinia pestis*	0

### Proteins involved in bacterial virulence

Out of 56 unique proteins, 30 matched significantly to known virulence-associated proteins from other Gram-negative and Gram-positive pathogenic bacteria in MVirDB (Table 3).

**Table 3:** Genes involved in bacterial virulence

Mutant	Locus	Number of hits	Lowest E-value	Protein	Location
Eis041	NT01EI_0943	250	0	esaC	Outer Membrane
Eis110	NT01EI_1332	91	0	eseL	Extracellular
Eis171	NT01EI_3723	22	0	Magnesium-translocating P-type ATPase	Cytoplasmic Membrane
Eis192	NT01EI_3147	10	0	Hypothetical protein	Unknown
Eis037	NT01EI_2355	192	9.13E-144	eseJ	Extracellular
Eis038	NT01EI_1334	267	3.95E-121	eseM	Extracellular
Eis001	NT01EI_1281	6	1.97E-95	NAD-dependent malic enzyme (NAD-ME)	Cytoplasmic
Eis195	NT01EI_0376	5	8.13E-87	Anaerobic C4-dicarboxylate transporter DcuA	Cytoplasmic Membrane
Eis173	NT01EI_3265	221	8.89E-61	Acyltransferase/AMP-dependent synthetase and ligase protein family	Cytoplasmic Membrane
Eis185	NT01EI_3386	5	1.74E-56	TRAP transporter DctM subunit	Cytoplasmic Membrane
Eis107	NT01EI_0475	11	2.34E-56	DEAD box containing helicase	Cytoplasmic
Eis230	NT01EI_3769	250	1.16E-46	Phosphoglycerate transporter family protein	Cytoplasmic Membrane
Eis028	NT01EI_0277	8	4.26E-43	Transposase, IS4 family protein	Unknown
Eis175	NT01EI_3774	34	6.93E-41	IS1 transposase	Unknown
Eis184	NT01EI_0419	8	2.71E-40	RNA methyltransferase TrmH family, group 3	Cytoplasmic
Eis176	NT01EI_0962	144	9.04E-38	esaT	Cytoplasmic Membrane
Eis157	NT01EI_0144	250	4.66E-29	Twin-arginine translocation protein subunit TatB	Cytoplasmic Membrane
Eis068	NT01EI_0448	2	4.13E-26	Polyprenyl synthetase	Cytoplasmic
Eis155	NT01EI_0725	9	4.05E-12	Transcriptional regulator FruR	Cytoplasmic
Eis086	NT01EI_1237	4	9.61E-12	N-acetylmuramoyl-L-alanine amidase AmiA	Unknown
Eis006	NT01EI_0085	40	9.81E-12	ATP-dependent DNA helicase Rep	Cytoplasmic
Eis235	NT01EI_3289	20	2.44E-10	Diaminopimelate decarboxylase	Cytoplasmic
Eis055	NT01EI_2314	22	7.18E-08	Prophage lambda integrase	Cytoplasmic
Eis207	NT01EI_1817	59	1.13E-07	Spermidine/putrescine transport system permease protein PotB	Cytoplasmic Membrane
Eis080	NT01EI_0981	10	1.52E-06	Hypothetical protein	Cytoplasmic
Eis222	NT01EI_0768	44	1.67E-06	Hypoxanthine phosphoribosyltransferase	Cytoplasmic
Eis018	NT01EI_0377	4	1.96E-06	Aspartate ammonia-lyase	Cytoplasmic
Eis011	NT01EI_3690	9	2.98E-04	ABC transporter, periplasmic amino acid binding protein	Periplasmic
Eis131	NT01EI_2157	21	9.55E-03	Hypothetical protein	Unknown
Eis223	NT01EI_1086	16	9.91E-03	Extracellular solute-binding protein, family 5	Unknown

### Safety and vaccine efficacy of mutants in catfish

**Fig. 3:**
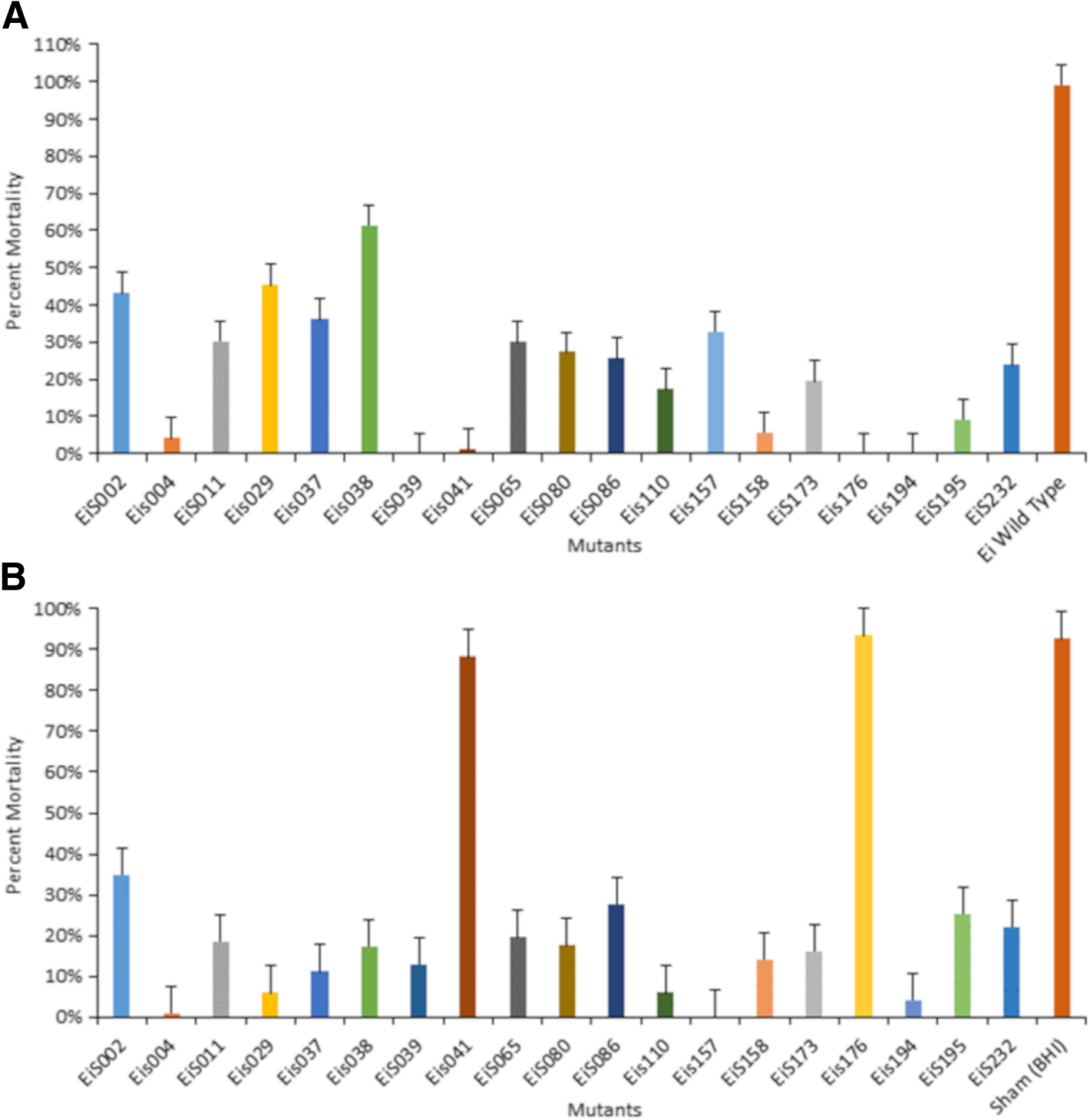
Virulence and efficacy of the transposon insertion mutants in channel catfish fingerlings. Percent mortalities and mutant names are indicated on the Y and X axis, respectively. Channel catfish fingerlings were infected with mutant strains to determine virulence and vaccinate the fish (**a**). After 21-days of post vaccination, fish were infected with wild-type *E. ictaluri* 93–146 to determine the efficacy of vaccination (**b**)

Safety testing of transposon-derived mutants showed that all mutant strains were attenuated significantly compared to wild-type control (*p* < 0.05). *Ei*s::*004, Ei*s::*039, Ei*s::*041, Ei*s::*176,* and *Ei*s::*194* caused less than 5% mortality, while mutants, *Ei*s::*110, Ei*s::*158,* and *Ei*s::*195* caused less than 10% mortality (Fig. 3a). We noticed that mutants with gene products assigned to cytoplasm caused lower mortalities compared to those found in outer membrane or extracellular regions. Interestingly, proteins with unknown location (*Ei*s::*194* and *Ei*s::*039*), did not show any mortalities in channel catfish. Mutants *Ei*s::*002, Ei*s::*011, Ei*s::*029, Ei*s::*037, Ei*s::*038, Ei*s::*065, Ei*s::*080, Ei*s::*086, Ei*s::*157, Ei*s::*173,* and *Ei*s::*232* were attenuated but caused over 20% mortality.

*Edwardsiella ictaluri* wild-type challenge of catfish 21 days post vaccination indicated that all mutants except *Ei*s::*041* and *Ei*s::*176* protected catfish significantly compared to sham vaccinated group (*p* < 0.05). *Ei*s::*004* and *Ei*s::*194* were the safest and most protective mutants, while *Ei*s::*157* protective but not safe (Fig. 3b). Although *Ei*s::*039, Ei*s::*041,* and *Ei*s::*176* were safe, they did not provide good immunization in catfish against *E. ictaluri* WT (Fig. 3).

## Discussion

The bioinformatics analyses of 56 unique genes with transposon insertions showed that more than half (54%) were potential virulence factors in other pathogenic bacteria. Among the virulence factors, Type III secretion system (T3SS), twin-arginine translocation pathway (Tat), and ATP-binding cassette transporter (ABC) seem to be important for *E. ictaluri* virulence and invasion of the channel catfish [25–27].

The functional gene ontology analysis with Blast2GO indicated that most of the proteins participate in cellular and metabolic networks in the biological process while their molecular functions frequently matched to binding and catalytic activity. The proteins located in extracellular regions are part of the signaling process or response to any stimulus sensing bacteria in the biological process. Although several proteins showed transporter activity, these proteins account for localization and are in the cytoplasm.

The subcellular locations predicted by PSORTb revealed that most of the identified proteins are found in the cytoplasm and cytoplasmic membrane. Although many well-known virulence proteins are located in the outer membrane or periplasm in Gram-negative bacteria, only three proteins of T3SS are located in extracellular space and outer membrane, and one of the ABC transporter proteins was found in the periplasmic space.

The host-pathogen interaction examined by HPIDB proved that many proteins have a high similarity to other virulence-associated proteins in different pathogenic bacteria including *Y. pestis*, *S. flexneri*, and *B. anthracis*. The pathogenic Gram-negative bacteria *Y. pestis* and *S. flexneri* share the same evolutionary lineage with *Edwardsiella sp.* in *Enterobacteriaceae* [28]. Thus, most of the virulence-associated proteins may have a similar role in *E. ictaluri*. Two T3SS effector proteins EseJ and EseM have a predicted interaction with the channel catfish ubiquitin-conjugating enzyme E2 (*XP_017323313*). These two T3SS-related effector proteins known as E3 ubiquitin ligase play an important role in manipulation of host ubiquitination pathways.

The interrupted genes *eseJ* (*Ei*s::*037*), *eseM* (*Ei*s::*038*), *esaC* (*Ei*s::*041*), *eseL* (*Ei*s::*110*), and *esaT* (*Ei*s::*176*) are part of T3SS, which are involved in export of proteins inside the host immune cells [29]. EsaC and EsaT are the structural membrane associated proteins of T3SS. *Ei*s::*41*, YscC ring-shaped structure protein in the outer membrane, is required for a stable oligomeric complex to shape a T3SS in the outer membrane [30]. YscT inner membrane-embedded component is located in the cytoplasm, which has extended and helical regions that may form membrane-bound subunits. Insertions in the T3SS related genes, *eseJ*, *eseM*, and *eseL*, have been recently identified to be T3SS dependent effector proteins [31]. EseJ, EseL, and EseM proteins share high similarities with *Salmonella* T3SS effector proteins Ssph2 and SlrP. They are involved in ubiquitination of proteins, an important process regulating inflammatory response in eukaryotes. As a part of novel E3 ligases (NELs) protein family, EseJ, E3 ubiquitin-protein ligase (SspH2), and EseL, a new class of E3 ubiquitin ligase, have a role in T3SS that provides a strategy to exploit host cell ubiquitin pathway [32]. EseM, T3SS leucine rich repeat protein (SlrP), is also required to form a complex ubiquitin ligase enzyme [33]. T3SS effector protein mutants *eseJ, eseM,* and *eseL*, and T3SS structural mutants *esaC* and *esaT* showed significantly decreased virulence. However, in comparison of protection level of those two main groups, T3SS structural proteins EsaC and EsaT have been caused less protection in catfish. Mutation in T3SS effector proteins provides better protection against pathogenic bacteria [34–36]. EseL has provided significant protection among other T3SS related effector proteins. T3SS effector proteins could contribute the bacterial survival inside host immune cells [37, 38].

Transport processes in bacterial cells through outer membrane and periplasmic space are linked to *E. ictaluri* metabolism to survive in the host environment as well as switching between various biochemical processes during different stages of ESC. *Ei*s::*157*, *tatB*, is located in the periplasmic space and is involved in the translocation of proteins including the components of respiratory complexes using a proton gradient as an energy source [39]. *tatB* mutant exhibited slow growth under low-iron conditions and observed a 10-fold decrease in *Legionella pneumophila* growth [40]. *Ei*s::*086*, *amiA*, is a Tat pathway dependent substrate encoding a cell wall amidase. Tat pathway mutant causes mislocalization of AmiA protein, preventing translocation in the periplasm [41, 42]. *Ei*s::*011*, ABC transporter periplasmic amino acid binding protein, is an important antigenic factor involved in adhesion and aspartate/glutamate transport in the microaerobic environment in *Campylobacter jejuni* [43]. *Ei*s::*207*, *potB*, encodes a protein associated with spermidine/putrescine transport system. Polyamines are mostly involved in stabilization of DNA for stress resistance, intracellular signaling processes, and swarming motility [44, 45]. Polyamines are also associated with the virulence in the intracellular pathogen *Salmonella enterica* [46]. *Ei*s::*002*, PTS system IIB component, is a cytoplasmic component of the major carbohydrate transport system highly conserved through bacteria [47]. PTS system participates in a variety of virulence mechanisms including biofilm formation, modulating the virulence gene expression, and regulating carbohydrate metabolism in pathogenic bacteria [48–50]. *Ei*s::*171*, magnesium-translocating P-type ATPase, is an inducible magnesium transport system when bacteria grow at the low concentration of magnesium. Although Mg^2+^ is not essential for virulence, it participates in many cellular activities as a cofactor [51]. Magnesium is the part of the regulatory network that regulates the virulence-associated mechanisms in *S. enterica* [52]. *Ei*s::*195*, *dcuA*, is encoded with aspartase in the same operon that is determined as an antiporter mechanism involved in the transport of aspartate under the anaerobic conditions [53]. DcuA function in the metabolic pathway under anaerobic conditions contributes the pathogenicity for the colonization in the lower oxygen level [54].

Pathogenic bacteria adapted different carbohydrate metabolism, which is activated by oxygen presence in the host environment. *Ei*s::*018*, aspartate ammonia-lyase, is involved in the production of fumarate activated specifically under anaerobic conditions while there is no available electron acceptor. Bacteria encodes aspartate ammonia-lyase to utilize alternative carbon sources in the host environment if there are no available carbon sources [55, 56].

Bacterial stress related proteins induce the protective mechanisms under a variety of stress conditions to protect the bacterial cell inside or outside of the host [57]. Universal stress protein A (UspA) in *Ei*s::194, is one of the stress proteins found in intracellular pathogenic bacteria. *uspA* expression reaches a high level when bacteria are exposed to heat, starvation, antimicrobial, and oxidative agents [58, 59]. UspA is a conserved protein that presents in Eubacteria, Archaea, plants, and fungi and the expression of UspA is triggered by exposure to oxidative agents in growth arrested cells [60–62]. UspA plays a significant role in the pathogenicity of bacteria, and *uspA* mutants are less virulent and sensitive to changes in the host environment. Mutation of *S. typhimurium C5 uspA* resulted in less virulence and more susceptibility to nutrient starvation oxidative agents [59]. In *Listeria monocytogenes*, *uspA* mutants were shown to have impaired activity in oxidative agent’s exposure to low pH conditions [58]. Deletion of *uspA* gene in *Acinetobacter baumannii* revealed that it has a significant role in protecting the bacteria from H_2_O_2_ and low pH [63].

*IscX* in *Ei*s::*S039*, acts as a regulator for the Fe-S (iron-sulfur) cluster, which encodes proteins essential for cell activities [64]. FeS assembly protein IscX (YfhJ) is a part of the iron-sulfur cluster (ISC) mediated FeS cluster, which is a small acidic protein that binds IscC and Fe, and acts as a Fe donor in FeS cluster [65, 66]. ISC mediated FeS biogenesis is involved in survival of bacteria that face with iron starvation and oxidative stress. In *S. flexneri*, ISC mutants were less invasive and cannot form plaques on Henle cells monolayers [67]. ISC transcriptional regulator *iscR* mutant in *Pseudomonas aeruginosa* caused more susceptibility to oxidative agents and a significant decrease in virulence [68]. The importance of ISC system in bacterial virulence has been emphasized in different studies. However, limited information is known about the role of IscX in bacterial virulence.

Hypothetical protein in *Ei*s::*004* is located in the cytoplasm. There is no available information about the function of this hypothetical protein in any virulence related mechanisms. However, decreased virulence and significant protection against ESC revealed that *Ei*s::*004* mutant could be considered as a vaccine candidate for live attenuated vaccine development.

## Conclusions

In summary, these results showed that random transposon mutagenesis in the *E. ictaluri* genome resulted in colonies with delayed growth on complex solid media, and many of the disrupted genes have important functions and potentially contribute to *E. ictaluri* virulence. Fish experiments showed that *Ei*s::*004, Ei*s::*039, Ei*s::*041, Ei*s::*110, Ei*s::*158, Ei*s::*176, Ei*s::*194,* and *Ei*s::*195* mutants were significantly attenuated, and *Ei*s::*004* and *Ei*s::*194* provided good immunization in catfish.

## Methods

### Bacterial strains, plasmids, and growth conditions

Bacterial strains and plasmids used in this work are listed in Table 4. *Edwardsiella ictaluri* 93–146 carrying pAK*gfplux*1 [69] was grown at 30 °C using brain heart infusion (BHI) broth and agar plates (Difco, Sparks, MD). *Escherichia coli* SM10λ*pir* donor strain carrying p*MAR2xT7* [20] was grown at 37 °C using Luria-Bertani (LB) broth and agar plates (Difco). Antibiotics were added to the culture medium at the following concentrations: ampicillin (100 μg/ml), colistin (12.5 μg/ml), and gentamicin (12.5 μg/ml).

**Table 4:** Bacterial strains and plasmids

Strain or plasmid	Description	Source
*Escherichia coli*		
SM10λ*pir*	km^r^; *thi; thr; leu; tonA; lacY; supE; recA;*::RP4–2-Tc::Mu; λ*pir* R6K	[75]
*Edwardsiella ictaluri*		
93–146	wild-type; pEI1; pEI2; Col^r^	[11]
Plasmids		
p*MAR2xT7*	R6K replicon; *Himar I*; T7 promoters; Amp^r^; Gen^r^	[20]

### Construction of transposon insertion library

Transposon insertion library was constructed by conjugation using the donor *E. coli* SM10λ*pir* carrying p*MAR2xT7* and the recipient *E. ictaluri* 93–146 wild type (WT) containing pAK*gfplux*1 [69]. Transposon insertion mutants were selected on selective BHI agar plates containing 100 μg/ml of ampicillin, 12.5 μg/ml of gentamicin, and 25 μg/ml colistin. Various sizes of gentamicin resistant transposon insertion colonies were observed on the selective BHI plates, and 250 smallest colonies compared to normal colony size were cultured in the BHI broth with colistin and gentamicin at 30 °C for 2 days. Finally, bacterial stocks were prepared in 20% glycerol and stored at − 80 °C freezer.

### Transposon end mapping

Genomic DNA was isolated from the frozen *E. ictaluri* transposon insertion mutants using the heat denaturation method. Briefly, 100 μl frozen culture were added in 1 ml ddH_2_O and mixed well. Bacteria were collected by centrifugation and water was removed completely. After dissolving the bacterial pellet in 100 μl ddH_2_O, each sample was transferred to 200 μl PCR tubes and tubes were incubated at 100 °C for 10 min by using an Applied Biosystems 2720 Thermal Cycler (Life Technologies, Grand Island, NY). Samples were mixed well by vortexing, and bacterial cell debris was pelleted by centrifuging at 14,000 rpm for 5 min. The supernatant containing the genomic DNA was used as template in subsequent PCR reactions. Single primer PCR was performed by using a transposon-specific R1 primer (5`-CCGTATGCCCAACTTTGTATAGA-3`) to amplify the transposon end and flanking bacterial DNA [70]. Before sequencing, the PCR products were cleaned by using ExoSAP-IT for PCR Product Cleanup (Affymetrix, Santa Clara, CA). Sequencing was conducted at Eurofins MWG Operon LLC (Huntsville, AL) using a transposon-specific nested R3 primer (5`- TCTCGGCTTGAACGAATTGTT-3`).

### Bioinformatics analyses

Transposon sequence removal and sequence trimming based on sequence quality scores were done by using the Sequencher DNA sequence analysis software v4.10.1 (Gene Codes Corp., Ann Arbor, MI). Trimmed sequences were searched against the available *E. ictaluri* 93–146 genome [29] by using basic local alignment search tool (Blast) at the National Center for Biotechnology Information (NCBI) for gene identification. Using the GI numbers, a FASTA file containing all protein sequences were downloaded from the Batch Entrez database of NCBI and used for downstream analysis. Gene Ontology (GO) annotation, visualization, and metabolic and cellular processes were determined by using Blast2GO [71] at the cut-off level 2. Subcellular localization of proteins was predicted by using PSORTb version 3.0.2 [72]. *E. ictaluri* proteins involving in host-pathogen interactions were identified by using the Host-Pathogen Interaction Database (HPIDB) at the cut-off level 0.0001. Bacterial proteins interacting with channel catfish proteins were determined at the cut-off level 0.00001, at identity filter 50% in bacterial proteins, and 70% in channel catfish proteins. [73]. The potential *E. ictaluri* virulence proteins were identified using the Microbial Virulence Database (MVirDB) at the cut-off level 0.5 [74]. TA sequence frequencies in the entire *E. ictaluri* genome, open reading frames, and genes with transposon insertion were calculated using CLC genomics workbench 11.0.1 (Qiagen, Redwood City, CA).

### Safety and vaccine efficacy testing of mutants in catfish

Specific pathogen free (SPF) channel catfish was obtained from the fish hatchery of the College of Veterinary Medicine at Mississippi State University. All fish experiments were conducted under a protocol approved by the Institutional Animal Care and Use Committee at Mississippi State University (protocol number 12–042). In vivo experiments were conducted using catfish infection model to test 19 mutants. Briefly, four-month-old pathogen free channel catfish (11.58 ± 0.23 cm, 15.29 ± 0.95 g) were stocked at a rate of 20 fish/tank into 40 L tanks and maintained at 26 ± 2 °C throughout the experiment. Each transposon mutant, positive (*E. ictaluri* wild-type), and negative (BHI) controls were assigned to three or four tanks randomly. Catfish were challenged/vaccinated by immersion exposure using transposon mutants or wild type (3.09 × 10^7^ CFU/ml of water) using published procedures [15]. Catfish mortalities were recorded for 21 days. After 21 days of the first vaccination, both vaccinated, and sham-vaccinated catfish were infected with *E. ictaluri* wild type by immersion exposure (3.27 × 10^7^ CFU/ml of water). Catfish mortalities were recorded for two weeks.

### Statistical analysis

We used SPSS V25 (IBM Corp., Armonk, NY) to conduct statistical analysis. For each strain, mean percent mortalities were calculated and arcsine-transformed. The one-way analysis of variance at significance level 0.05 was conducted using the “Univariate” function, in which strains were independent and arcsine-transformed mortalities were dependent variables. Because our data included different sample sizes, and variances were not equal, Games-Howell post hoc test was selected to identify significant differences between mutants and wild type or mutants and sham vaccinated group in virulence and efficacy experiments, respectively.

## Abbreviations

ESC: Enteric septicemia of catfish
HPIDB: Host-Pathogen Interaction Database
IACUC: Institutional Animal Care and Use Committee
MVirDB: Microbial Virulence Database
PSORTb: Subcellular Localization Prediction Tool
T3SS: Type III Secretion System
UspA: Universal Stress Protein A
